# Correlations between components of the immune system

**DOI:** 10.12688/f1000research.54487.1

**Published:** 2021-11-22

**Authors:** Yehudit Shabat, Yaron Ilan

**Affiliations:** 1 Hadassah University Hospital, Jerusalem, Jerusalem, Israel, Israel

**Keywords:** complex systems; immune memory; information transfer; immune response; randomness

## Abstract

**Background: **No evidence of the possibility to alter a constituent of the immune system without directly affecting one of its associated components has been shown yet.

**Methods: **A schematic model was developed in which two triggers, fasting and splenectomy, were studied for their ability to affect the expression of cell membrane epitopes and the cytokine secretion of out-of-body autogeneic and syngeneic lymphocytes.

**Results**: Fasting decreased expression of CD8 and CD25 and increased TNFα levels. The effect of splenectomy as a trigger was investigated in non-fasting mice by comparing splenectomized and non-splenectomized mice. An increase in the CD8 expression and in TNFα, IFNg, and IL10 secretion was noted. The effect of splenectomy as a trigger was investigated in fasting mice by comparing splenectomized and non-splenectomized mice. Splenectomy had a significant effect on expression of CD25 and CD4 CD25 and on secretion of TNFα, IFNg, and IL10. To determine the effect of keeping the cells in an out-of-body location on the expression of lymphocyte epitopes, tubes kept on top of the cages of the fasting mice were compared with tubes kept on top of empty cages. A significant change in the CD8 expression was noted. To determine the effect of keeping cells in an out-of-body location on cytokine secretion, tubes kept on top of cages were tested for cytokine levels. A significant decrease was noted for the secretion of TNFα and IFNg.

**Conclusions: **The data obtained from this study characterized a system for induction of correlations between two components of the immune system without a transfer of mediators. The study showed that a mouse could affect cells at a distance and alter the expression of surface markers and cytokine secretion following two types of triggers: fasting and/or splenectomy. Thus, an out-of-body correlation can be induced between two components of the immune system.

## Abbreviations

IFNγ: interferon gamma

IL10: interleukin 10

OL: an independent control laboratory

TGFβ: transforming growth factor beta

TNFα: tumor necrosis alpha

## Introduction

Most living systems contain information. The more complex the system is, the higher the probability that it carries information. The immune system, similar to other biological systems, may optimize functionality, but it does not necessarily have perfect structure or symmetry. The irregularity that underlines some of the pathways of this system provides the opportunity to apply the concepts of physics to this biological system. ^1^

The correlations between components of the immune system always involve reactions mediated by direct association between different elements of this system. ^2^ Current paradigms of immune crosstalk between cells or at a subcellular molecular level are based on direct contact or on transfer of mediators in both health and disease. ^3^^–^^5^ These elements serve as links for relocating associations between constituents of the immune system. No evidence for correlations between components of the immune system that alter a constituent of this system without directly affecting one of its associated parts has been reported.

The aim of the study was to develop a system in which a correlation exits between two separate components of the immune system without either a direct interaction or a transfer of mediators.

## Methods

### Animals

A total of 24 Male C57BL/6 healthy mice (11–12 weeks old, 30 gr.) were obtained from Harlan Laboratories (Jerusalem, Israel) and maintained in the Animal Core of the Hadassah-Hebrew University Medical School. Mice were administered standard laboratory chow and water *ad libitum* and kept in a 12-hour light/dark cycle. Mice were administered standard laboratory chow and water *ad libitum* and kept in a 12-hour light/dark cycle. Animal experiments were carried out according to the guidelines of the Institutional Committee for Care and Use of Laboratory Animals and with the committee’s approval. All efforts were made to ameliorate any suffering of animals by using mild anesthesia.

### Experimental groups

Six groups of mice ( Table 1) were studied, with four mice per group in each of the four experiments. Number of animals was determined based on statistical analysis. Data on all mice in each of the studies is included in the *Underlying data*. ^21^ Groups were selected based on the different parameters tested with the appropriate controls.

**Table 1.** Experimental groups (n = 4 for each group).

Group	Mouse in cage	Splenectomy	Fasting	Autogeneic cells on top of cage	Syngeneic cells on top of cage
A	+	+	+	+	-
B	+	+	+	-	+
C	+	+	-	+	-
D	+	+	-	-	+
E	+	-	+	-	+
F	+	-	-	-	+
G/H	-	-	-	-	-

The mice in groups A and B underwent splenectomy followed by fasting. Each mouse was kept in a cage with a tube containing its own autogeneic (group A) or syngeneic (group B) lymphocytes on top. The mice in groups C and D underwent splenectomy but did not fast, and they were kept in cages with tubes containing autogeneic (group C) or syngeneic (group D) lymphocytes on top. The mice in groups E and F did not undergo splenectomy and were kept in cages with a tube containing syngeneic lymphocytes on top. The mice in group E fasted, while that in group F did not fast. Tubes marked G and H contained lymphocytes harvested from syngeneic donors and were kept on top of empty cages. Controls were used for each of the experiments based on the parameter tested. Studies were repeated in an outside laboratory for verification of the effect. Studies were conducted in accordance with the Arrive guidelines.

### Study design

The described system enabled us to study two types of triggers for the associations between two constituents of the immune system. Mice underwent splenectomy using standard procedure ^6^ or fasting (21-24 hours). Each mouse was kept in a separate cage for the duration of the experiment. Autogeneic or syngeneic splenocytes were prepared from splenectomized mice and kept for 24 hours in a tube on top of the cage at a distance of 10-20 cm from the mouse itself. Control cells were kept in a tube placed on top of the cages containing either non-splenectomized or non-fasting naïve mice or were kept on top of empty cages without a mouse. In splenectomized mice, we tested the effect of splenectomy alone or splenectomy combined with fasting on either autogeneic or syngeneic cells. In naïve, non-splenectomized mice, we tested the effect of fasting on syngeneic cells. The effects of these triggers on the associations between immune components were examined by determining the expression of surface markers and cytokine secretion by the out-of-body lymphocytes. The study was replicated three times and for each study four mice per group were analyzed (marked 11A, 11B, and 11C). For epitope parameters, the experiment was repeated for the fourth time by an independent control laboratory (marked OL) for several of the parameters. Cytokine study was performed once in the controlled laboratory.

### Flow cytometry

Splenocytes were isolated as described previously. ^7^ Flow cytometry was performed on splenocytes resuspended in 1 mL of Flow Cytometry Staining (FACS) buffer. Cells were stained with diluted antibodies (50 μL/sample). Cells were examined using FACS at times 0 and 24 hours for the expressions of epitopes, CD3, CD4, CD8, CD25, FoxP3, and NK1.1, on the lymphocytes (antibodies by BD Biosciences). Flow cytometry was performed using an LSR-II flow cytometer (BD Biosciences, DeNovo software). The following subsets were analyzed: CD4, CD25, CD4 CD25, Foxp3, CD4 Foxp3, CD4 CD25 Foxp3, CD4 CD25 Foxp3, CD8, CD8 Foxp3, CD8 CD25, CD8 CD25 Foxp3, CD8 CD25 Foxp3, CD3, NK1.1, and CD3 NK1.1. Full data of the study can be accessed from the *Underlying data* ^21^ section.

### Cytokine measurement

One set of mice marked OL (n = 4) was analyzed in the control laboratory for serum levels of IFNγ, TNFα, IL10, and TGFβ. Determination of IFNγ, TNFα, and IL10 in supernatants was performed concurrently, using Flow Cytomix (Thermo Fisher Scientific (eBioscience)). TGFβ levels were determined separately using ELISA Quantikine (MB100B) (R&D Systems). Cytokine levels were determined according to the manufacturer’s instructions.

### Statistical analysis

The Mann-Whitney SPSS test (software version 1.0) was performed for each experiment. Data of all individual mice in all studies is provided in the *Underlying data*. ^21^ Only parameters that showed significant changes in two experiments or more, including changes in opposing directions, were used for the final analysis.

## Results

The effect of fasting and/or splenectomy on promoting correlations between immune systems was studied by determining the alterations in expressions of cell membrane epitopes and in cytokine secretion by out-of-body autogeneic and syngeneic lymphocytes.

**Figure 1.**
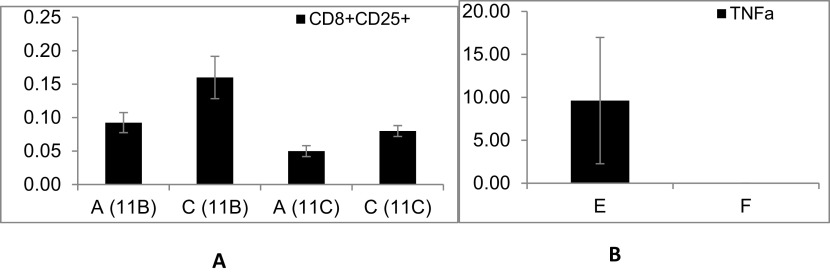
The effect of fasting as a trigger for inducing associations between components of the immune system of splenectomized mice was determined by comparing the autogeneic lymphocytes from groups A (fasting) vs. C (non-fasting); there was a significant effect on the expression of CD8 and CD25 epitopes. The effect of fasting as a trigger on splenectomized mice was determined by comparing the syngeneic lymphocytes kept in tubes on top of the cages of the mice in groups B (fasting) vs. D (non-fasting). The effect of fasting as a trigger for an association that may alter the epitope expression and/or cytokine secretion of out-of-body lymphocytes kept in tubes on top of cages was determined by comparing the syngeneic lymphocytes of the mice in groups E (fasting) vs. F (non-fasting); there was a significant effect on the TNFα secretion.

The effect of fasting as a trigger for altering the association that can modify epitope expression on out-of-body lymphocytes kept in tubes on top of cages was investigated in splenectomized mice. The effect on autogeneic cells was determined by comparing groups A (fasting) and C (non-fasting). Figure 1A shows the effect of fasting on autogeneic lymphocytes; decreased expression of CD8 and CD25 was found in the mice in group A compared with mice in group C in experiments 11B (p = 0.02) and 11C (p = 0.019). Fasting exerted a significant effect on the cytokine secretion by the out-of-body syngeneic lymphocytes in non-splenectomized mice. Figure 1B shows a significant increase in the TNFα levels (p = 0.047) when comparing group E (fasting) to group F (non-fasting, the TNFα levels were undetectable in-group F). The data suggested that correlations can be induced between components of the immune system after fasting in splenectomized and non-splenectomized mice targeting both out-of-body autogeneic and syngeneic lymphocytes. Fasting had no effect on the syngeneic lymphocytes kept in a tube on top of the cages of splenectomized mice, as indicated by comparing groups B (fasting) and D (non- fasting).

**Figure 2.**
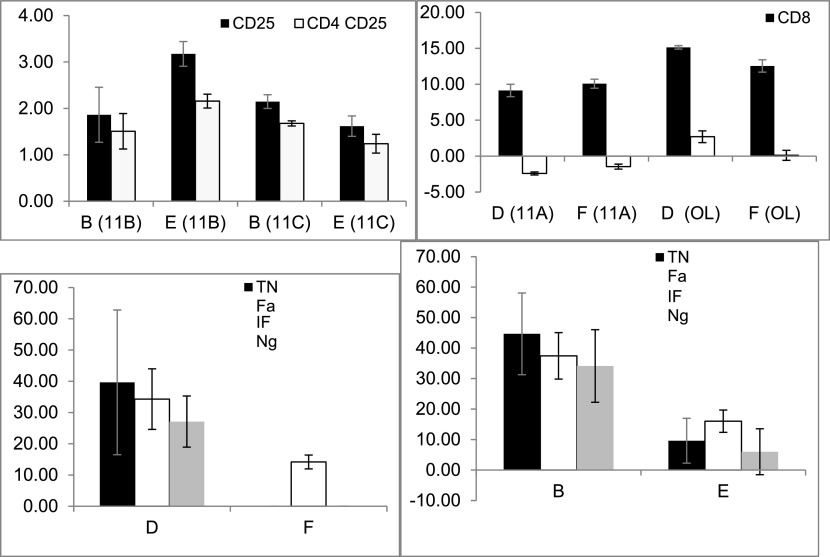
The effect of splenectomy as a trigger for an association that may alter the epitope expression and/or cytokine secretion of out-of-body lymphocytes kept in tubes on top of cages was determined by comparing the syngeneic lymphocytes kept in the tubes on top of the cages containing the non-fasting mice from groups D (splenectomized) vs. F (non-splenectomized). The effect was significant for CD8 expression and for TNFα, IFNγ, and IL10 secretion. The effect was also determined by comparing the syngeneic lymphocytes that were kept in tubes on top of the cages containing fasting mice from groups B (splenectomized) vs. E (non-splenectomized). It was significant for the expressions of CD25 and CD4 CD25, and for TNFα, IFNγ, and IL10 secretion.

The effect of splenectomy as a trigger for altering the association that can modify epitope expression and/or cytokine secretion from out-of-body syngeneic lymphocytes kept in tubes on top of cages was investigated in non-fasting mice by comparing groups D (splenectomized) and F (non-splenectomized). Figure 2A shows a significant increase in the CD8 expression (between time points 0 and 24 h) in experiments 11A (p = 0.021) and OL (p = 0.021) in-group D compared with that in-group F; however, the changes in CD8 expression in experiment 11A were opposite to those in experiment OL. The secretion of three cytokines, TNFα (p = 0.014), IFNγ (p = 0.021), and IL10 (p = 0.013), significantly increased in group D compared with group F, as shown in Figure 2B.

The effect of splenectomy as a trigger for modifying the epitope expression and/or cytokine secretion of out-of-body syngeneic lymphocytes was investigated in fasting mice by comparing groups B (splenectomized) and E (non-splenectomized). Figure 2C shows that splenectomy had a significant effect on several parameters, as indicated by comparing the two groups in experiments 11B and 11C; however, the effects in group B were opposite to the effects in group E. The expression of CD25 (p = 0.02 and p = 0.03 for 11B and 11C, respectively) and CD4 CD25 (p = 0.021 and p = 0.034, for 11B and 11C, respectively) differed significantly between groups B and E. The secretion of TNFα (p = 0.021), IFNγ (p = 0.02), and IL10 (p = 0.02) was significantly higher in group B compared with that in group E, as shown in Figure 2D. The results suggested that a link can be induced between components of the immune system after splenectomy in fasting and non-fasting mice in syngeneic lymphocytes. No significant differences were observed when assessing differences between the effects on out-of-body autogeneic vs. syngeneic cells; these differences were determined by performing a group A (autogeneic) vs. B (syngeneic) comparison of the lymphocytes harvested from the fasting-splenectomized mice and by performing a group C (autogeneic) vs. D (syngeneic) comparison of the non-fasting splenectomized mice. The results suggested that the associations made for autogeneic cells did not differ significantly from that made for syngeneic cells.

**Figure 3.**
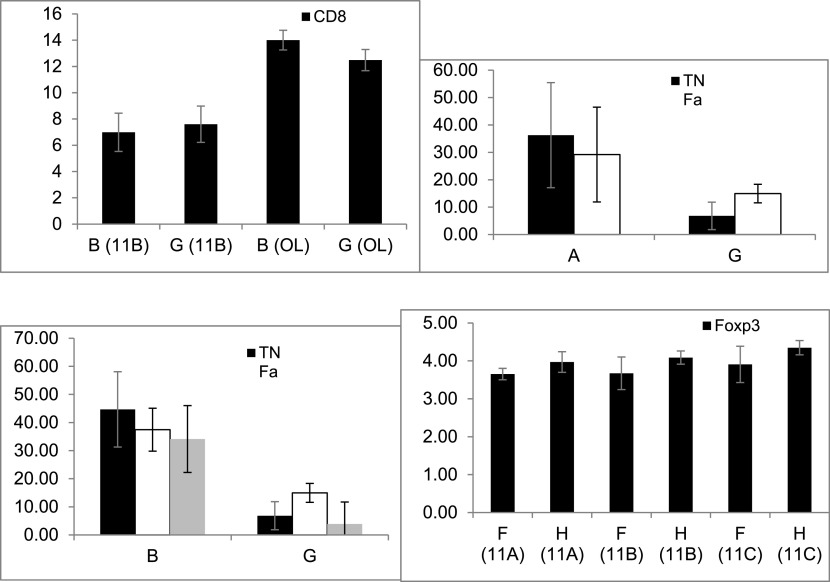
The effect of the presence of a mouse in a cage on altering the expression of membrane epitopes and cytokine secretion was determined by analyzing the differences between the tubes from all groups and the tubes kept on empty cages (G and H). The effect was significant for CD8 expression when comparing groups B and G (3A); for the secretion of TNFα and IFNγ when comparing groups A and G (3B); for the secretion of TNFα, IFNγ, and IL10 when comparing groups B and G (3C); for the expression of Foxp3 when comparing groups F and H (3D); for TNFα, IFNγ, and TGFβ secretion when comparing groups C and H (3E, 3F); for IFNγ, TNFα, and IL10 secretion when comparing groups D and H (3G); and for TNFα secretion when comparing groups F and H (3H).

To determine the effect of keeping the cells in an out-of-body location on the expression of lymphocyte epitopes, tubes kept on top of the cages of the fasting mice from groups A, B, and E were compared with tubes kept on top of empty cages (G), as shown in Figure 3. A significant change in the CD8 expression was noted by comparing groups B and G in 11B (p = 0.034) and OL (p = 0.043), as shown in Figure 3A; however, the changes in group B were opposite to those in group G. No significant effect was observed when comparing groups A and G.

To determine the effect of keeping cells in an out-of-body location on cytokine secretion, tubes kept on top of cages were tested for cytokine levels. A significant decrease was noted for the secretion of TNFα (p = 0.021) and IFNγ (p = 0.028) between groups A and G, as shown in Figure 3B. Similarly, a significant decrease was noted between groups B and G for the secretion of TNFα (p = 0.021), IFNγ (p = 0.021), and IL10 (p = 0.018), as shown in Figure 3C. No significant difference was observed between groups E and G for the epitope expression or cytokine secretion.

To determine the effect of keeping cells in an out-of-body location on the expression of lymphocyte epitopes, tubes kept on top of cages containing the non-fasting mice from groups C, D, and F were compared with tubes kept on top of empty cages (H). The Foxp3 expression significantly increased between groups F and H in all three experiments, as shown in Figure 3D (for 11A, p = 0.083; for 11B, p = 0.081; and for 11C, p = 0.083; p = 0.006 for all three; this parameter was not tested in OL). No significant difference was observed when comparing groups C vs. H and groups D vs. H.

To determine the effect of keeping cells in an out-of-body location for cytokine secretion, tubes kept on top of cages were assessed for cytokine levels. A significant decrease in the secretion of IFNγ, TNFα, and TGFβ was observed when comparing groups C and H, as shown in Figure 3E (p = 0.020) and Figure 3F (p = 0.43). A significant decrease in the secretion of IFNγ, TNFα, and IL10 was observed between groups D and H, as shown in Figure 3G (p = 0.021). A significant increase in the TNFα secretion was observed between groups F and H, as shown in Figure 3H (p = 0.047). The data suggested that the presence of a fasting or non-fasting mouse in a cage affects its connections with syngeneic cells.

## Discussion

The present study presents measurements of parameters over a set of mutually unbiased states, which demonstrated an ability to induce a correlation between components of the immune system through an indirect effect in an isolated system. The described experiment showed that a mouse could affect cells at a distance and alter the expression of surface markers and cytokine secretion following two types of triggers: fasting and/or splenectomy. In the described laboratory setting only the measured observables, which are the result of this effect, can be detected. The effects were compared for cells kept on top of cages containing mice that did not undergo a splenectomy and did not fast with cells kept on empty cages.

In biological systems, different levels of associated states may be present. These levels might depend on the degree of comparison and/or correlation between donors and recipients that act simultaneously on various components of the immune system. ^3^^–^^5^ In the present schematic model, a correlation between two systems, a cell, or other component of the immune system, allows for the recovery of a new state of a lymphocyte or cytokine-secreting cell.

The lymphocyte expression of cell surface markers and cytokine secretion involved different pathways. ^7^^–^^10^ The “classical” transportation of immune signals is based on messages delivered by molecules (e.g., chemokines) secreted by different subsets of cells that transmit a direct signal to a target cell or to a subcellular organelle (e.g., receptor) through physical contact or a messenger molecule. The present data demonstrated that in an isolated system, a correlation can be induced independent of a direct interaction. The observed effects cannot be explained by classical immunology, and are, therefore, suggestive of a correlation-dependent phenomenon in the described system.

The effect that underlines the outcome observed in the present study on autogeneic cells can be explained by an inherent association between two components of the immune system, suggesting an undefined historical correlation between the two parts of the system. At the same time, the effects observed on syngeneic cells may imply a wave-dependent phenomenon between foreign components of the immune system.

The fidelity obtained in the current schematic model shows excellent adherence of properties of correlations when targeting the expression of cell membrane epitopes and cytokine secretion of lymphocytes. The results from each mouse in the described system suggest an inherent pattern that repeats itself. Each attempt for separate mice is viewed independent of all others. The use of a standard trigger, fasting or splenectomy, enables our protocol to succeed without filtering of results, and the effects are fairly reliable. The experiments were repeated in an independent laboratory, further supporting the feasibility of setting up isolated conditions, under which these effects can be measured.

The lack of effects for all parameters in all experiments (see *Underlying data* ^21^) and changes in opposing directions for some of the measured observables may result from the multiple confounding factors that act simultaneously, which are hard to control, or from the disordered nature of the immune pathways and networks. These are inherent to any biological system. ^11^^–^^14^ Nonetheless, the out-of-body correlation was significant for several of the investigated parameters in repeated experiments. For some of the observables, the changes were small and could be claimed to be a result of intra-test variability. However, each mouse was tested in a separate cage, and can be viewed as independent of the other mice. Only parameters that significantly changed in two separate experiments were used for the final data analysis.

The results described in the present study may support the presence of built-in memory in an isolated system, which is mandatory for such correlations to occur. Although the memory in immune systems is based on the direct delivery of mediators or messenger molecules secreted by immune cells, ^15^^,^^16^ a “wave type of memory” is required at both the transmitting and receiving sites for such a correlation to occur. This type of an immune memory may explain some of the observations in the present study.

The effects observed in the present study may occur at a cellular or subcellular level and may act in accordance with theories that apply some of the principals of quantum physics to biological systems. ^11^^,^^17^^–^^19^ A model using a non-natural computer-brain interface to induce an out-of-body effect has been reported, which showed non-invasive information transfer between the brains of different species. ^20^ A translation of the intention of a human volunteer to stimulate the rat brain motor area responsible for tail movement has also been demonstrated. The data suggested the feasibility of a computer-mediated brain–brain interface that can link the neural functions between two biological entities.

The system described here provides a robust model for biological applications of correlations in immunology and biology. The model demonstrated here could also be used as an elementary constituent of a wave-type repeater in other biological systems. The state implemented in this protocol is based on complex systems, which enables modifying the surface markers and cytokine secretion of lymphocytes. Even with a relatively low success probability, this system can be scaled to more complex biological systems.

Optimal function of the immune system may require an alliance between classical and correlative immunology. The present data shed light on several, not fully understood, biological phenomena, including inter-immune cells interactions, brain–immune component connections, environmental impacts, genotype-phenotype interfaces, and organism-host interactions. By enabling diagnostic and therapeutic procedures to be performed on out-of-body autogeneic or syngeneic tissues or organisms, these concepts may be applicable to the development of improved methods for diagnosis and treatment of immune-associated disorders.

Study limitations include the use of the defined model and extrapolations to other biological systems may require further studies.

## Data availability

### Underlying data

Dryad: Correlations between components of the immune system, https://doi.org/10.5061/dryad.prr4xgxn3. ^21^

This project contains the following underlying data:

- LDB_EM_Supplementary_File_1.5.xls

Data are available under the terms of the Creative Commons Zero “No rights reserved” data waiver (CC0 1.0 Public domain dedication).

### Reporting guidelines

PLoS Biol: ARRIVE checklist for ‘Correlations between components of the immune system’, https://doi.org/10.1371/journal.pbio.3000411. ^22^
