# Supplementary material for: Correlations between components of the immune system
Source: F1000Res. 2024 Mar 12;10:1174. Originally published 2021 Nov 22. [Version 3] doi: 10.12688/f1000research.54487.3 (PMC11019305; doi:10.12688/f1000research.54487.3)
Supplement: Peer review history

# Reviewer report: Reviewer response for version 3

**DOI:** 10.5256/f1000research.163303.r255016

**Published:** 2024-04-15

**Authors:** Rohit Malik

**Affiliations:** 1 Department of Pharmacology, Gurugram Global College of Pharmacy, SRM Modi Nagar College of Pharmacy, SRMIST, Delhi-NCR Campus, Ghaziabad, Gurugram, Haryana, India

The Authors updated the manuscript as per the suggestions.

Is the work clearly and accurately presented and does it cite the current literature?

No

If applicable, is the statistical analysis and its interpretation appropriate?

Partly

Are all the source data underlying the results available to ensure full reproducibility?

Partly

Is the study design appropriate and is the work technically sound?

No

Are the conclusions drawn adequately supported by the results?

No

Are sufficient details of methods and analysis provided to allow replication by others?

No

Reviewer Expertise:

Ethnopharmacology and Natural Product Chemistry

I confirm that I have read this submission and believe that I have an appropriate level of expertise to confirm that it is of an acceptable scientific standard.

# Reviewer report: Reviewer response for version 2

**DOI:** 10.5256/f1000research.156314.r245655

**Published:** 2024-02-28

**Authors:** Rohit Malik

**Affiliations:** 1 Department of Pharmacology, Gurugram Global College of Pharmacy, SRM Modi Nagar College of Pharmacy, SRMIST, Delhi-NCR Campus, Ghaziabad, Gurugram, Haryana, India

The manuscript necessitates significant revisions:

 1) The Abstract's Results section is unclear, as it merely presents outcomes without clearly articulating the determination process as stated.

 2) Reconstruct the Conclusion section of the Abstract to align with your findings.

 3) Add the complete abbreviations as mentioned in the manuscript.

 4) Major revisions are needed in the Introduction section to justify the aspects of your study adequately.

 5) Why n=4 in animals groupings, instead of n=6 Justify.

 6) Insert a graphical representation of the research protocol with the grouping of animals and day-wise plan.

 7) Cross-check the sequence of references in the manuscript.

 8) Why was SPSS used instead of GraphPad Prism?

 9) Regenerate the figures to include standard deviation (SD), standard error of the mean (SEM), degrees of freedom (Df) values, comparisons among all groups, and R-squared values for a clearer understanding of the results.

 10) In the discussion part, mention the findings with figure numbers when compared with previous studies.

 11) Add the conclusion part to the manuscript.

Is the work clearly and accurately presented and does it cite the current literature?

No

If applicable, is the statistical analysis and its interpretation appropriate?

Partly

Are all the source data underlying the results available to ensure full reproducibility?

Partly

Is the study design appropriate and is the work technically sound?

No

Are the conclusions drawn adequately supported by the results?

No

Are sufficient details of methods and analysis provided to allow replication by others?

No

Reviewer Expertise:

Ethnopharmacology and Natural Product Chemistry

I confirm that I have read this submission and believe that I have an appropriate level of expertise to state that I do not consider it to be of an acceptable scientific standard, for reasons outlined above.

# Response

**Published:** 2024-03-01

**Authors:** Yaron Ilan

**Affiliations:** Hadassah-University Medical Center, Israel

**Response to reviewers**

1. *The Abstract's Results section is unclear, as it merely presents outcomes without clearly articulating the determination process as stated.*

**Response:** The paragraph was rewritten for improved clarity.

*2) Reconstruct the Conclusion section of the Abstract to align with your findings.*

**Response:** We accepted the comment, and the paragraph was revised.

*3) Add the complete abbreviations as mentioned in the manuscript.*

**Response:** Corrected

*4) Major revisions are needed in the Introduction section to justify the aspects of your study adequately.*

**Response:** The *Introduction* section was revised for improved clarity.

*5) Why n=4 in animals groupings, instead of n=6 Justify.*

**Response:** The study design determined the number of animals to enable significance. We have revised the relevant section for improved accuracy.

*6) Insert a graphical representation of the research protocol with the grouping of animals and day-wise plan.*

**Response:** We have revised Table 1 and added Table 2 with a graphical representation of the research protocol as suggested.

*7) Cross-check the sequence of references in the manuscript.*

**Response:** Corrected.

*8) Why was SPSS used instead of GraphPad Prism?*

**Response:** The method was recommended by one of the reviewers.

*9) Regenerate the figures to include standard deviation (SD), standard error of the mean (SEM), degrees of freedom (Df) values, comparisons among all groups, and R-squared values for a clearer understanding of the results.*

**Response:** A new set of figures is included with the SD. We have revised the relevant paragraphs in the results to include the statistical data.

*10) In the discussion part, mention the findings with figure numbers when compared with previous studies.*

**Response:** Corrected as suggested.

*11) Add the conclusion part to the manuscript.*

**Response:** A conclusion section was added as suggested.

# Reviewer report: Reviewer response for version 2

**DOI:** 10.5256/f1000research.156314.r210454

**Published:** 2023-10-06

**Authors:** Suprabhat Mukherjee

**Affiliations:** 1 Integrative Biochemistry & Immunology Laboratory, Department of Animal Science, Kazi Nazrul University, Asansol, West Bengal, India

Authors have addressed all the queries.

Is the work clearly and accurately presented and does it cite the current literature?

Yes

If applicable, is the statistical analysis and its interpretation appropriate?

Partly

Are all the source data underlying the results available to ensure full reproducibility?

Yes

Is the study design appropriate and is the work technically sound?

Yes

Are the conclusions drawn adequately supported by the results?

Yes

Are sufficient details of methods and analysis provided to allow replication by others?

Partly

Reviewer Expertise:

NA

I confirm that I have read this submission and believe that I have an appropriate level of expertise to confirm that it is of an acceptable scientific standard.

# Reviewer report: Reviewer response for version 1

**DOI:** 10.5256/f1000research.57980.r197558

**Published:** 2023-09-11

**Authors:** Biswadeep Das

**Affiliations:** 1 School of Biotechnology, Kalinga Institute of Industrial Technology, KIIT Deemed to Be University, Bhubaneswar, Odisha, India

The manuscript depicts the correlation of different components of the immune system in fasting and splenectomy scenarios. Though the topic is moderately interesting, and in context to metabolism and immune system cross-talk, the overall manuscript and the methods are inadequate to justify its claims.

 Major  issues:

1. The introduction is too small and does not involve the rationale behind selecting such a research work. Need to elaborate significantly.
2. Methods are the major limitation: The selection of mice need to be well defined. Flow cytometry is very subtly written. The authors say that, "The following subsets were analyzed: CD4, CD25, CD4 CD25, Foxp3, CD4 Foxp3, CD4 CD25 Foxp3, CD4 CD25 Foxp3, CD8, CD8 Foxp3, CD8 CD25, CD8 CD25 Foxp3, CD8 CD25 Foxp3, CD3, NK1.1, and CD3 NK1.1."
  However, this is just a random statement. How to classify the different immune cells without using a backbone flow cytometry, LCA? First major classification need to be done, followed by subset analysis. Where are the raw files of flow cytometry? What about autofluorescence and compensation? How did u confirm the expression of these markers using experimental and instrumental controls?
3. Western blot need to be done to confirm the existence of the markers in the given population. It is missing.

Results:

1. No flow cytometry graph or quadrant analysis. Just a mere bar diagram does not prove the analysis. Western blot has to be shown for all the markers as confirmed by flow cytometer.

In view of these facts, I cant be more positive and have to opt for rejection, unless all these issues are addressed.

Is the work clearly and accurately presented and does it cite the current literature?

No

If applicable, is the statistical analysis and its interpretation appropriate?

Partly

Are all the source data underlying the results available to ensure full reproducibility?

Partly

Is the study design appropriate and is the work technically sound?

No

Are the conclusions drawn adequately supported by the results?

Partly

Are sufficient details of methods and analysis provided to allow replication by others?

No

Reviewer Expertise:

Biomedical genetics and diganostics

I confirm that I have read this submission and believe that I have an appropriate level of expertise to state that I do not consider it to be of an acceptable scientific standard, for reasons outlined above.

# Response

**Published:** 2023-09-17

**Authors:** Yaron Ilan

**Affiliations:** Hadassah-University Medical Center, Israel

1. The *Introduction *section was revised as proposed for better clarity. The relevant references were inserted.
2. A supplementary file is included with all the data of all experiments. The relevant section in the Methods was revised for better clarity.
3. Western blots were not conducted in the present study and are subject to future studies.
4. The data of all studies is included in the supplementary file. The graphs were not included as multiple analyses were conducted in these experiments.

# Reviewer report: Reviewer response for version 1

**DOI:** 10.5256/f1000research.57980.r144783

**Published:** 2022-09-02

**Authors:** Suprabhat Mukherjee

**Affiliations:** 1 Integrative Biochemistry & Immunology Laboratory, Department of Animal Science, Kazi Nazrul University, Asansol, West Bengal, India

- Approval/reference number of the meeting of the Institutional Animal Ethics Committee for the Mice related experiments is required.
- Figures need to be revised.
- Names of the cytokines should be formatted correctly.
- Please perform ANOVA following a post-hoc test to test the difference amongst the cytokine level.
- Immunofluorescence data may be added to strengthen the data of FACS and ELISA.

These changes are necessary to improve the manuscript.

Is the work clearly and accurately presented and does it cite the current literature?

Yes

If applicable, is the statistical analysis and its interpretation appropriate?

Partly

Are all the source data underlying the results available to ensure full reproducibility?

Yes

Is the study design appropriate and is the work technically sound?

Yes

Are the conclusions drawn adequately supported by the results?

Yes

Are sufficient details of methods and analysis provided to allow replication by others?

Partly

Reviewer Expertise:

Immunology of Inflammatory and Infectious diseases

I confirm that I have read this submission and believe that I have an appropriate level of expertise to confirm that it is of an acceptable scientific standard, however I have significant reservations, as outlined above.

# Response

**Published:** 2023-09-17

**Authors:** Yaron Ilan

**Affiliations:** Hadassah-University Medical Center, Israel

1. Approval details were added.
2. All figures were replaced with a more explicit version.
3. Corrected.
4. The Mann-Whitney SPSS test (software version 1.0) was used for revised statistical analysis.
5. These were not performed in the current study and are subject to future experiments.
